# Knowledge, perceived risk, and attitudes towards COVID-19 protective measures amongst ethnic minorities in the UK: A cross-sectional study

**DOI:** 10.3389/fpubh.2022.1060694

**Published:** 2023-01-13

**Authors:** Erica Jane Cook, Elizabeth Elliott, Louisa Donald, Alfredo Gaitan, Gurch Randhawa, Sally Cartwright, Muhammad Waqar, Chimeme Egbutah, Ifunanya Nduka, Andy Guppy, Nasreen Ali

**Affiliations:** ^1^School of Psychology, University of Bedfordshire, Luton, United Kingdom; ^2^Public Health Luton, Luton Borough Council, Luton, United Kingdom; ^3^Institute for Health Research, University of Bedfordshire, Luton, United Kingdom

**Keywords:** COVID-19, knowledge, risk, health beliefs and attitudes, ethnicity, communication

## Abstract

**Background:**

Minority ethnic groups are at increased risk of COVID-19 related mortality or morbidity yet continue to have a disproportionally lower uptake of the vaccine. The importance of adherence to prevention and control measures to keep vulnerable populations and their families safe therefore remains crucial. This research sought to examine the knowledge, perceived risk, and attitudes toward COVID-19 among an ethnically diverse community.

**Methods:**

A cross-sectional self-administered questionnaire was implemented to survey ethnic minority participants purposefully recruited from Luton, an ethnically diverse town in the southeast of England. The questionnaire was structured to assess participants knowledge, perceived risk, attitudes toward protective measures as well as the sources of information about COVID-19. The questionnaire was administered online *via* Qualtrics with the link shared through social media platforms such as Facebook, Twitter, and WhatsApp. Questionnaires were also printed into brochures and disseminated *via* community researchers and community links to individuals alongside religious, community and outreach organisations. Data were analysed using appropriate statistical techniques, with the significance threshold for all analyses assumed at *p* = 0.05.

**Findings:**

1,058 participants (634; 60% females) with a median age of 38 (IQR, 22) completed the survey. National TV and social networks were the most frequently accessed sources of COVID-19 related information; however, healthcare professionals, whilst not widely accessed, were viewed as the most trusted. Knowledge of transmission routes and perceived susceptibility were significant predictors of attitudes toward health-protective practises.

**Conclusion/recommendation:**

Improving the local information provision, including using tailored communication strategies that draw on trusted sources, including healthcare professionals, could facilitate understanding of risk and promote adherence to health-protective actions.

## 1. Introduction

The Coronavirus disease 2019 (COVID-19) outbreak, caused by the severe acute respiratory syndrome coronavirus-2 (SARS-CoV-2) (1) has created devastation with high morbidity and mortality rates worldwide (2). COVID-19 has not affected all sections of the UK's diverse population equally, with ethnic minority communities shown to be disproportionally impacted (3, 4). Data reported by the Office for National Statistics revealed that black ethnic groups are around 4.3 times whilst Bangladeshi and Pakistani ethnic groups are around 1.8 times more likely to have a COVID-19 related death when compared to the rest of the population, even when adjusting for socio-demographic characteristics, self-reported health and disability (5). These differences can be explained by a complex range of interrelated demographic, socio-economic and cultural factors, including poverty and deprivation, overcrowded housing, working in occupations with a higher risk of COVID-19 exposure (frontline care workers, key worker occupations with public-facing roles), need to use public transportation, health service access alongside historic racism (6, 7).

Currently, there is no treatment available that can act specifically against the SARS-CoV-2 infection (8). The successful development and implementation of vaccines is so far our most effective defence against SARS-CoV-2 infection, shown to deliver robust protection against severe disease, hospitalisation and mortality from all variants of the Coronavirus present in the UK (9). However, the UK's minority ethnic population, who despite being at higher risk of contracting COVID-19 and experiencing poorer health outcomes, continue to have had a disproportionally lower uptake of the COVID-19 vaccine compared to their white British counterparts (10, 11), further exacerbating existing health inequalities. The outbreaks prevention and control measures have been and continue to be an important strategy to control the spread of COVID-19 in the UK. Government guidance has encouraged the UK population to take individual responsibility by adopting preventative personal health actions, including hand washing, ventilation, social distancing, mask-wearing, respiratory hygiene (covering mouth and nose while coughing or sneezing) and limiting close contacts (12). However, the success of such efforts remains dependent on the public adherence to these recommendations (13).

The extent to which an individual will engage in preventive behaviours is affected by many factors. Knowledge, perceptions of risk and susceptibility toward the COVID-19 infection (2), and attitudes toward preventive behaviours (2, 14), are revealed to be important determinants. Moreover, the accessibility of information and trust in those sources have also been shown to be influential in the adoption of preventive health practises among ethnic minority communities (13, 15, 16). As such, understanding how ethnically diverse communities access information and their trust in these sources will provide useful insight on how to best deliver important key public health messages.

This research aimed to examine knowledge, perceived risk, and attitudes toward COVID-19 among an ethnically diverse community in the UK. This will provide useful insights to inform the planning and delivery of COVID-19 related health promotion initiatives that can seek to empower ethnically diverse communities to protect themselves and their families (17). This study is part of a wider community engagement programme “Talk Listen Change” (TLC) (also incorporating qualitative sub-studies) which sought to understand views on the disproportionate rate of COVID-19, the reasons for and impacts of this to tackle related health inequalities and co-develop solutions.

## 2. Method

### 2.1. Study design and participants

A cross-sectional community survey was conducted in January 2021 and March 2021 during the peak of the second wave of COVID-19. Luton is an industrial town located in the Southeast of England with a population of just under 220,000. The population in Luton is ethnically diverse and is one of the only three towns in the UK to have a white British population of < 50%. Luton has a large Asian/Asian British and black/black British population which is four and six times the national average, respectively (18). Luton experiences high rates of deprivation compared to other parts of the UK (19), with nine output areas in the top ten per cent most deprived areas in the country (20). The inclusion criteria for the survey required participants to be 16 years and older, living in Luton, UK and identifying as a member of the Pakistani, Bangladeshi, Indian, black African or Caribbean community. These ethnic groups were purposively targeted as they have been disproportionately impacted by COVID-19 (3, 6).

### 2.2. Data collection

An online survey was placed onto Qualtrics and was advertised *via* a wide range of social network online platforms, including Facebook (local community groups, Luton County Council), Twitter, WhatsApp groups and Instagram. Hashtags, photos, and links were used to further encourage engagement. There was also a local press release alongside interviews and discussions with several radio stations that advertised the study hourly in both English and Urdu. Posters were also disseminated across Luton in local shops, places of worship, community centres and local food banks with QR code links provided.

Questionnaires were also printed into brochures and disseminated *via* community links to individuals, religious, community and outreach organisations. The research team also trained and supported a team of TLC community researchers who became “super recruiters” and disseminated questionnaires through their networks using snowballing techniques. The TLC community researchers resided in Luton and were from the religious, cultural, and linguistic backgrounds of the communities of interest. They also held an in-depth knowledge of the local community, formal and informal organisations and networks and were subsequently able to engage with the “less visible” members of the community. TLC community researchers also, where necessary, translated and completed the community survey on behalf of participants who had poor English language fluency.

### 2.3. Sample size

Luton currently hosts a total population of 213,500, whereby Asian/Asian British (61,000), black/black British (19,000), and mixed ethnic groups (*n* = 8,300) account for a total of 41.4% (*n* = 88,300) of the total population (21). As there is insufficient evidence to determine the factor/s of interest we used a conservative estimate of 50% of the population having the factor of interest (22). An online sample size calculator (23) was used and based on a 40% response rate, 5% precision or margin of error, 50% proportion with a 95% confidence interval of the population of interest (88,300) a sample size of 957 was deemed to provide sufficient power for the study.

### 2.4. Questionnaire design

A questionnaire was designed by the research team and covered socio-demographic characteristics, knowledge regarding COVID-19, perceived risk factors, perceived likelihood of serious events related to COVID-19 and attitudes toward COVID-19 protective measures (Supplementary material 1).

#### 2.4.1. Socio-demographic characteristics and health

Participants provided self-reported socio-demographic data for age, gender, ethnicity (Indian, Pakistani, Bangladeshi, African, Caribbean, mixed ethnic background, other); religious faith (Christian which included Church of England, Catholic, Protestant and all other Christian denominations, Buddhist, Hindu, Jewish, Muslim, Sikh, none, other). Participants were asked to report their highest level of education (no qualifications, GCSE or equivalent, A-level, or equivalent, first degree (e.g., BSc, BA), higher degree (e.g., MSc, MA) or “other”. Full postcode was also requested which was then matched to the Index of Multiple Deprivation (IMD) 2015 (24) score using Geo Convert software (25). All IMD measures were divided into ten deprivation deciles, with each decile comprising 10% of the population of England with a lower decile indicating increasing deprivation. Changes to employment status since the pandemic and current living arrangements (living alone, with immediate family, living with extended family) was also captured. Participants were also asked if they, their parents, and/or their grandparents were born in the UK (Yes/No) and if they or anyone in their household have an existing chronic health condition e.g., chronic lung disease, diabetes, cardiovascular disease, chronic renal or liver disease (Yes/No).

#### 2.4.2. Knowledge (symptoms/routes of transmission)

Knowledge of symptoms was assessed by asking participants if the symptoms presented were symptomatic of COVID-19. A total of 20 symptoms (common or less common) as listed by the World Health Organisation and the Centre for Disease Control and Prevention (26) were presented to participants. Each correct response received one point with zero points for unanswered or incorrect answers. The total knowledge score was calculated based on the number of valid/correct answers (27), with a maximum total score of 20, and a minimum of 0. Internal consistency of this scale was found to be good (*a* = 0.90).

Knowledge of COVID-19 transmission routes was assessed by asking participants to indicate to what extent they believed that (1) close contact with an infected person who has symptoms, (2) close contact with an infected person even if they aren't showing symptoms of infection and (3) contact with surfaces an infected person has touched were potential transmission routes to infection (28). Each correct response received one point with zero points for unanswered or incorrect answers, with a minimum of 0 and a maximum total score of 3 (*a*= 0.76).

#### 2.4.3. Perceived risk factors of contracting COVID-19 and poor health outcomes due to COVID-19

Participants were provided with ten risk factors that have been documented in the literature as contributing to putting ethnically diverse populations at increased risk of contracting COVID-19. The risk factors included; “types of employment (e.g., front line health and social care workers, work in public-facing services)” (6, 29–31), “lack of confidence to raise concerns about safety in the workplace” (6, 32), “use of public transport to get to work/other” (6, 32), “living in more densely populated areas” (6, 32), “low income or financial insecurity” (30, 32), “living in overcrowded accommodation” (6, 32), “living in multi-generational housing” (6, 31, 32), “education and understanding about how to reduce personal risk of contracting COVID 19” (29–31), “individual behaviours (e.g. not washing hands, wearing a face mask, and keeping at least 2 m away from others)” (33) and “English literacy and proficiency” (29). Participants were asked to state to what extent they believed each of the ten risk factors contributed to putting ethnically diverse populations at risk of contracting COVID-19. Responses were recorded on a five-point scale ranging from (1) strongly disagree to (5) strongly agree, with a maximum total score of 50 and a minimum of 10 (*a* = 0.86).

Participants were then provided with a further set of ten “risk factors” and were then asked to rate to what extent they perceived each factor contributed to putting ethnically diverse populations at increased risk of having poorer health outcomes as a result of contracting COVID-19. Risk factors included; “having a pre-existing health condition (such as having underlying conditions like diabetes, hypertension, cardiovascular disease and obesity)” (6, 29, 31), “mental health issues/illness” (6), “poor access to healthcare services” (31), “lack of trust of NHS services and health care treatment” (6, 34), “stigma relating to fear of diagnosis or death” (6), “low income or financial insecurity” (30), “living in areas with high deprivation” (31, 32), “vitamin D deficiency” (35, 36), “education and understanding about symptom recognition and when to access health services for COVID-19” (6, 29, 31, 34), “English literacy and proficiency” (29, 31, 34). Responses were recorded on a five-point scale ranging from (1) strongly disagree to (5) strongly agree with a maximum total score of 50 and a minimum of 10 (*a* = 0.85).

#### 2.4.4. Perceived likelihood of events related to COVID-19

Participants were provided with eight events such as “*you will be infected”* and “*you will have to go to hospital if you get infected”* (28) and they were asked to rate the likelihood of these events happening to them on a six-point Likert scale ranging from (1) extremely unlikely to (6) already happened. Items that were not relevant were given a score of 0. This scale a minimum of 0 and a maximum total score of 48, whereby the higher the score the more susceptible an individual feels in respect of events related to COVID-19 (*a* = 0.85).

#### 2.4.5. Attitudes toward COVID-19 protective measures

Participants were presented with a total of nine protective measures e.g., “*wearing a facemask”*, “*washing your hands frequently”*, “*avoiding public spaces, gatherings, and crowds”* (37) and were asked to indicate to what extent they believed each measure would keep them safe from COVID-19. Responses were rated on a five-point Likert scale ranging from (1) not effective at all to (5) extremely effective. The maximum total score was 45, with a minimum of 9, with a higher score representing more positive attitudes toward protective measures (*a* = 0.86).

#### 2.4.6. Frequency of use and validation of information sources

Participants were provided with 15 “communication” sources, examples included “*twitter*”, “*WhatsApp*”, “*family members*” and they were asked to rate the frequency of using these sources to access information about COVID-19 on a five-point Likert scale ranging from (1) never to (5) multiple times per day. Scores ranged from a minimum of 15 and a maximum of 75 (*a* = 0.85). Participants were then asked to indicate to what extent they validated these sources to ensure that they provide accurate information about COVID-19 with responses recorded on a four-point scale ranging from (1) not at all to (4) completely. Scores ranged from a maximum of 60 and a minimum of 15 (*a* = 0.89).

To examine differences between sources of information and socio-demographic and health-related differences, the sources were placed into four categories which included (1) social media (Twitter, Facebook, Instagram, and WhatsApp), (2) social networks (family members, friends, co-workers), (3) mainstream media (national and local radio, national and local TV) and healthcare professionals (doctors/healthcare professionals).

Participants were also asked three questions which assessed to what extent participants determined the accuracy of the sources they used to access COVID-19 related information. Using a five-point frequency scale ranging from “strongly disagree (1)” to “strongly agree (5),” we asked participants “how do you ensure that information you have accessed about COVID-19 is accurate' with three statements provided. Statements included (1) “Information is accessed from a reputable well-known source”, (2) “I compare information I access with other reliable sources to ensure it is accurate” and (3) “I access information objectively to determine the information I read is presented in a balanced, reasonable and unbiased manner”. The total score had a maximum of 15, a minimum of 3, with a higher score indicating higher validation of information sources (*a* = 0.89).

### 2.5. Data analysis

Preliminary analysis of the data using Shapiro-Wilk tests and visual inspection indicated the data was largely normally distributed, consequently where possible parametric statistics were used. Descriptive statistics of frequencies, means, ranges and standard deviations were calculated to describe all participants' socio-demographic characteristics. A series of one-way Univariate Analyses of Variance (ANOVAs) were conducted to explore possible differences between groups based on socio-demographic factors in terms of knowledge of symptoms and transmission routes, perceived susceptibility of COVID-related events alongside attitudes towards COVID-19 protective measures. *Post hoc* analyses (Tukey's HSD) were performed for multiple comparisons where appropriate.

Hierarchal multiple regression analysis was conducted on the whole sample to examine whether participants' socio-demographic factors along with knowledge of COVID-19 symptoms and transmission routes and perceived levels of susceptibility toward COVID-19 could predict their attitudes toward COVID-19 protective measures. Only the significant correlates identified were entered into the regression analysis. Pairwise deletion (available-case analysis) was used to handle missing data. All statistical tests were completed using IBM SPSS Version 26; two-tailed significance was assumed at *p* < 0.05.

### 2.6. Ethical considerations

This study was conducted according to the guidelines of the Declaration of Helsinki and approved by the Research Ethics Committee of the University of Bedfordshire (protocol code IHREC957; 18th December 2020). All participants were informed about the purpose and procedure of the study before participation. Informed consent (written) was obtained from all participants involved in the study. Participants who took part using the online survey were presented with a landing page which provided research participation information and a consent statement. Participants who agreed and continued the survey were deemed to have consented, and participants who selected “no” were directed to the end of the survey with their data excluded. Written informed consent was obtained for all participants who completed the paper survey. The survey was only available in English; however, TLC Community Researchers administered the survey to anyone who was unable to read the questionnaire. All data were collected and analysed anonymously.

## 3. Results

### 3.1. Sample characteristics

A total of 1,200 questionnaires were printed and disseminated across the local community through the TLC community researchers. 767 questionnaires were returned, which yielded a total response rate of 64% for the paper survey. In addition, 291 responses were received using the online survey platform which provided a total sample of 1,058 participants who were included in this study. The overall sample as presented in Table 1 was representative of the ethnically diverse population in Luton (38). Participants were aged between 16 and 87 with a median age of 38 (IQR, 22). Around 42% of all participants stated that they had a first degree (e.g., BSc) and/or a higher degree (e.g., MSc) with 12% of participants stating that they had no formal qualifications. Around 15% of participants stated that they live with a chronic disease condition with 24% disclosing that they currently live with someone who has a chronic health condition.

**Table 1:** Descriptive characteristics of the sample (*N* = 1,058).

**Variable**		**Count (%)**
**Age group**	16–30 years	361 (34.1%)
	31–40 years	200 (18.9%)
	41–50 years	244 (23.1%)
	51–65 years	178 (16.8%)
	66–100 years	56 (5.3%)
	Missing	19 (1.8%)
**Gender**	Male	414 (39.1%)
	Female	634 (59.9%)
	Other/prefer not to say	10 (1.0%)
**Education Level**	No formal qualifications	123 (11.6%)
	GCSE or equivalent	178 (16.8%)
	A-Level or equivalent	233 (22.0%)
	First Degree (e.g., BSc, BA)	294 (27.8%)
	Higher degree (e.g., MSc, MA)	147 (13.9%)
	Other	83 (7.8%)
**Ethnicity**	Indian	132 (12.5%)
	Pakistani	477 (45.1%)
	Bangladeshi	176 (16.6%)
	Black African	91 (8.6%)
	Black Caribbean	75 (7.1%)
	Mixed: white and black Caribbean	9 (0.9%)
	Mixed: white and black African	6 (0.6%)
	Mixed: white and Asian	21 (2.0%)
	Any other ethnic background	61 (5.3%)
	Missing	10 (0.9%)
**Religious faith**	Christian	154 (14.6%)
	Hindu	53 (5.0%)
	Muslim	728 (68.8%)
	Sikh	48 (4.5%)
	No religion	38 (3.6%)
	Other (Jewish, Jainism, other not provided)	37 (3.6%)
**Born in UK**	Participant	564 (53.3%)
	Parents	190 (18%)
	Grandparents	17 (1.6%)
	No-one	257 (24.3%)
**Index Multiple Deprivation (Decile)**	1—Most deprived	12 (1.1%)
	2	162 (15.3%)
	3	117 (11.1%)
	4	98 (9.3%)
	5	109 (10.3%)
	6	24 (2.3%)
	7	44 (4.2%)
	8	53 (5.0%)
	9	14 (1.3%)
	10—Least deprived	0
**Live with Chronic disease**	Yes	155 (14.7%)
	No	870 (82.2%)
	Missing	33 (3.1%)
**Live with someone with a chronic disease**	Yes	252 (23.8%)
	No	652 (61.6%)
	Missing/Not relevant	154 (14.6%)

### 3.2. Knowledge of COVID-19

Participants had a good general awareness of the main symptoms of COVID-19, including the presence of a cough (*n* = 909; 86%), fever (*n* = 900; 85%) and loss of ability to smell (*n* = 890; 94%) and taste (*n* = 921; 87.1%). However, less common symptoms such as headaches and muscle/joint aches, alongside more serious symptoms (i.e., seizures and loss of consciousness) were less well understood as symptoms of COVID-19 (Supplementary Table 2). As presented in Table 2, differences in knowledge of symptoms varied by ethnicity [F_(1, 4)_=2.73, *p* = 0.028] (Table 2) whereby participants who self-identified as Indian had less knowledge of the symptoms compared to mixed and “any other' ethnic groups.

**Table 2:** Univariate Analyses of Variance (ANOVAs) of study variables by socio-demographic and knowledge, perceived risk and attitudes toward health protective actions.

**Variable**	**Knowledge**				**Likelihood of serious events**		**Attitudes toward health**	
							**protective actions**	
	**Symptoms**		**Transmission**					
	**Mean score** **(95% CI)**	***F*** **-value**	**Mean score** **(95% CI)**	***F*** **-value**	**Mean score** **(95% CI)**	***F*** **-value**	**Mean score** **(95% CI)**	***F*** **-value**
**Age group**								
16–30 years	9.46 (8.94–9.98)^a^	0.10	2.47 (2.37–2.56)^a^	0.15	27.97 (27.24–28.69)^a^	6.16^***^	35.43 (34.70–36.17)^a^	3.08^**^
31–40 years	9.36 (8.67–10.05)^a^		2.47 (2.33–2.59)^a^		27.91 (26.82–28.99)^ab^		36.25 (35.16–37.35)^ab^	
41–50 years	9.42 (8.79–10.06)^a^		2.51 (2.39–2.62)^a^		30.59 (29.63–31.55)^c^		35.92 (34.99–36.84)^ab^	
51–65 years	9.55 (8.73–10.26)^a^		2.52 (2.38–2.66)^a^		29.85 (28.69–31.00)^bcd^		37.05 (36.12–37.98)^ab^	
65 years+	9.80 (8.43–11.17)^a^		2.46 (2.21–2.72)^a^		29.34 (27.16–31.52)^abcd^		38.49 (36.77–40.21)^b^	
**Gender**								
Male	9.08 (8.61–9.55)^a^	3.55	2.52 (2.44–2.61)^a^	1.09	28.52 (27.79–29.24)^a^	2.08	35.79 (35.10–36.48)^a^	2.34
Female	9.68 (9.28–10.09)^a^		2.46 (2.39–2.54)^a^		29.21 (28.61–29.80)^a^		36.48 (35.92–37.03)^a^	
**Education level**								
No formal qualifications	9.82 (8.97–10.67)^a^	0.36	2.36 (2.20–2.52)^a^	3.46^*^	28.93 (27.64–30.22)^a^	1.62	36.66 (35.39–37.92)^a^	1.05
GCSE or equivalent	9.22 (8.44–9.99)^a^		2.42 (2.27–2.56)^ab^		27.89 (26.75–29.03)^a^		35.44 (34.32–36.57)^a^	
A-Level or equivalent	9.48 (8.79–10.16)^a^		2.47 (2.35–2.59)^ab^		29.05 (28.23–29.87)^a^		36.00 (35.10–36.91)^a^	
Degree/University	9.50 (9.05–9.94)^a^		2.60 (2.52–2.68)^b^		29.36 (28.62–30.10)^a^		36.46 (35.79–37.12)^a^	
**Ethnicity**								
Indian	8.80 (7.99–9.60)^a^	2.73^*^	2.59 (2.45–2.79)^a^	0.50	28.62 (27.44–29.79)^ab^	3.52^**^	37.11 (35.99–38.23)^ab^	3.04^*^
Pakistani	9.39 (8.97–9.82)^ab^		2.46 (2.38–2.55)^a^		29.47 (28.80–30.13)^a^		35.96 (35.31–36.61)^ab^	
Bangladeshi	10.09 (9.28–10.89)^ab^		2.48 (2.35–2.62)^a^		28.65 (27.59–29.72)^ab^		37.19 (36.22–38.17)^a^	
Black African/Caribbean	9.46 (8.68–10.25)^ab^		2.49 (2.35–2.63)^a^		27.31 (26.04–28.59)^b^		34.99 (33.89–36.10)^b^	
Mixed/Other	11.53 (9.41–13.64)^b^		2.50 (2.14–2.86)^a^		31.43 (29.53–33.33)^a^		34.76 (32.23–37.29)^ab^	
**Religious faith**								
Muslim	9.58 (9.21–9.95)^a^	1.77	2.46 (2.39–2.53)^a^	0.68	29.31 (28.76–29.86)^ac^	9.02^***^	36.12 (35.60–36.68)^a^	1.16
Christian	9.53 (8.71–10.35)^a^		2.53 (2.38–2.67)^a^		26.57 (25.20–27.93)^b^		35.55 (34.41–36.68)^a^	
Other (Sikh, Jews, Buddhist, Hindu, other)	8.75 (7.97–9.53)^a^		2.54 (2.41–2.68)^a^		29.74 (28.72–30.76)^bc^		36.79 (35.70–37.88)^a^	
**Employment**								
Paid employment	9.60 (9.22–9.99)^a^	0.38	2.56 (2.49–2.62)^a^	5.45^*^	29.49 (28.91–30.07)^a^	8.28^**^	36.28 (35.74–36.83)^a^	0.27
Other	9.40 (8.89–9.91)^a^		2.42 (2.32–2.52)^b^		28.09 (27.35–28.83)^b^		36.04 (35.32–36.77)^a^	
**Change in Employment**								
No change	9.51 (9.02–10.00)^a^	1.66	2.60 (2.45–2.61)^a^	1.00	29.25 (28.93–30.35)^a^	0.40	36.43 (35.32–36.65)^a^	0.61
Any change	10.04 (941–10.68)^a^		2.53 (2.49–2.71)^a^		29.64 (28.24–30.26)^a^		35.99 (35.52–37.35)^a^	
**Living status**								
Living alone	9.26 (8.22–10.32)^a^	0.17	2.46 (2.26–2.66)^a^	0.14	25.93 (24.12–27.74)^a^	8.29^***^	35.30 (33.83–36.76)^a^	3.51^*^
Living with immediate family	9.56 (9.21–9.90)^a^		2.51 (2.44–2.57)^a^		29.23 (28.72–29.74)^b^		36.15 (35.66–36.64)^ab^	
Living with extended family	9.45 (8.55–10.34)^a^		2.52 (2.37–2.68)^a^		29.40 (28.13–30.68)^b^		37.78 (36.57–39.00)^b^	
**Chronic health conditions**								
Yes	9.19 (8.34–10.04)^a^	1.54	2.48 (2.33–2.63)^a^	1.16	30.49 (19.23–31.76)^a^	7.87^**^	37.17 (36.10–38.25)^a^	3.31
No	9.72 (9.40–10.05)^a^		2.56 (2.50–2.61^a^		28.63 (18.13–29.12)^b^		36.01 (35.54–36.49)^a^	
**Live with someone with chronic health condition**								
Yes	9.44 (8.86–10.01)^a^	0.36	2.58 (2.47–2.68)^a^	0.68	29.53 (28.64–30.43)^a^	0.81	37.05 (36.21–37.89) ^a^	4.27^*^
No	9.65 (9.27–10.04)^a^		2.52 (2.46–2.59)^a^		29.04 (28.48–29.61)^a^		35.96 (35.41–36.51) ^b^	

Means sharing the same superscript letter are not significantly different from each other (p < 0.05), CI, confidence interval; ^*^p < 0.05, ^**^p < 0.01, ^***^p < 0.001.

Contact with surfaces an infected person has touched was least recognised as a route of transmission (*n* = 825; 78%) compared with close contact with an infected person with (*n* = 918; 87%) and without symptoms (*n* = 886; 84%) (Supplementary Table 3). The knowledge of COVID-19 transmission routes varied according to the participants' education level [*F*_(3, 971)_ = 3.46, *p* = 0.016] and employment status [*F*_(1, 1028)_ = 5.45, *p* = 0.002] (Table 2). It is important to note that participants not in paid employment were overrepresented by younger (aged 16–30 years) females who had lower levels of education (either no formal qualification or GCSE or equivalent) compared to those in paid employment.

### 3.3. Sources of information

A total of 79.8% (*n* = 844) participants stated that they currently own a computer, with a further 84.3% (*n* = 892) who stated that they currently own a smartphone (a phone that also has computer capabilities and access to the internet). The most regularly used sources to access information about COVID-19 included national TV, family members, WhatsApp, and friends, with the least used sources including local newspapers, Twitter, and healthcare professionals (Supplementary Table 4). Information sources were placed into four categories (1) social media, (2) social networks, (3) mainstream media and (4) healthcare professionals (Table 3).

**Table 3:** Frequency of sources used (by category) by participants to access information about COVID-19.

	***N***	**Min**	**Max**	**Mean**	**SD**
Social Media (Twitter, Facebook, Instagram, WhatsApp)	865	4.00	20.00	9.95	4.80
Social networks (family, friends, work colleagues)	873	3.00	15.00	9.20	3.55
Mainstream media (National and Local TV, National and Local radio)	862	4.00	20.00	10.83	4.47
Healthcare professionals	893	1.00	5.00	2.05	1.15

Participants use of social media varied by participants' age [*F*_(4, 846)_ = 4.56, *p* < 0.001], education level [*F*_(3, 805)_ = 11.81, *p* < 0.001] and employment status [*F*_(1, 848)_ = 4.48, *p* = 0.035]. Older participants (aged 65 years+), alongside those with lower levels of education and those not currently in employment were found to be significantly less likely to seek health information through this method. Further, participants who either disclosed that either they [*F*_(1, 853)_ = 4.81 *p* = 0.030] or someone they live with has an existing chronic health condition [*F*_(1, 757)_ = 9.88, *p* = 0.002] were shown to be more likely to access COVID-19 related information (Table 4).

**Table 4:** Univariate Analyses of Variance (ANOVAs) of study variables by socio-demographic and information seeking behaviour.

**Variable**	**Social media**		**Social networks**		**Mainstream media**		**Healthcare professionals**		**Validation of sources**	
	**Mean score (95% CI)**	***F*** **-value**	**Mean score (95% CI)**	***F*** **-value**	**Mean score (95% CI)**	***F*** **-value**	**Mean score (95% CI)**	***F*** **-value**	**Mean score (95% CI)**	***F*** **-value**
**Age group**										
16–30 years	10.60 (10.06–11.13)^a^	4.56^***^	9.10 (9.35–10.13)^a^	0.18	9.85 (9.37–10.33)^a^	8.26^***^	2.03 (1.90–2.15)^a^	1.74	11.54 (11.22–11.85)^a^	2.39
31–40 years	10.12 (9.35–10.89)^ab^		9.52 (9.22–10.41)^a^		11.05 (10.37–11.73)^b^		2.22 (2.03–2.40)^a^		11.83 (11.38–12.29)^a^	
41–50 years	9.86 (9.19–10.54)^ac^		9.17 (9.04–10.12)^a^		10.93 (10.34–11.53)^ab^		1.92 (1.77–2.07)^a^		11.3 (10.96–11.77)^a^	
51–65 years	9.21 (8.42–9.99)^bc^		9.42 (9.20–10.49)^a^		12.11 (11.33–12.89)^b^		2.14 (1.95–2.33)^a^		12.16 (11.75–12.57)^a^	
65 years+	7.82 (6.54–9.10)^c^		8.52 (8.33–10.70)^a^		12.39 (10.89–13.89)^b^		2.02 (1.66–2.38)^a^		11.13 (10.17–12.08)^a^	
**Gender**										
Male	10.23 (9.73–10.75)^a^	2.73	9.90 (9.52–10.28)^a^	1.48	11.15 (10.67–11.63)^a^	3.05	2.04 (1.92–2.16)^a^	0.01	11.71 (11.42–12.00)^a^	0.23
Female	9.69 (9.27–10.11)^a^		9.59 (9.25–9.91)^a^		10.60 (10.22–10.99)^a^		2.03 (1.94–2.13)^a^		11.62 (11.37–11.87)^a^	
**Education level**										
No formal qualifications	7.87 (7.12–8.63)^a^	11.81^***^	9.70 (8.91–10.49)^a^	0.69	10.25 (9.37–11.14)^a^	1.69	1.95 (1.77–2.14)^abc^	4.65^**^	10.53 (10.00–11.05)^a^	7.91^***^
GCSE or equivalent	9.37 (8.64–10.11)^ab^		9.38 (9.46–10.48)^a^		10.50 (9.80–11.21)^a^		1.92 (1.74–2.10)^ab^		11.25 (10.81–11.68)^ab^	
A-Level or equivalent	10.00 (9.34–10.66)^bc^		9.97 (9.46–10.48)^a^		10.36 (9.78–10.94)^a^		1.89 (1.74–2.04)^abc^		11.76 (11,40–12.12)^b^	
Degree/University	10.83 (10.30–11.35)^c^		9.74 (9.36–10.13)^a^		11.07 (10.59–11.55)^a^		2.21 (2.09–2.34)^c^		11.95 (11,64–12.27)^b^	
**Ethnicity**										
Indian	10.23 (9.29–11.17)^a^	1.04	9.28 (8.82–10.22)^a^	1.68	12.12 (11.21–13.02)^a^	4.43^**^	2.26 (2.04–2.48)^a^	2.27	11.93 (11.44–12.43)^ab^	2.34
Pakistani	9.89 (9.44–10.33)^a^		9.31 (9.59–10.29)^a^		10.38 (9.98–10.79)^b^		2.04 (1.93–2.15)^a^		11.40 (11.12–11.68)^ab^	
Bangladeshi	9.93 (9.14–10.73)^a^		9.12 (8.96–10.23)^a^		10.53 (9.85–11.22)^b^		1.90 (1.73–2.07)^a^		11.68 (11.21–12.14)^ab^	
Black African/Caribbean	9.99 (9.12–10.85)^a^		9.60 (8.96–10.23)^a^		11.38 (10.52–12.23)^ab^		2.16 (1.29–2.28)^a^		12.23 (11.82–12.79)^ab^	
Mixed/Other	8.15 (6.34–9.95)^a^		8.19 (6.43–9.93)^a^		11.82 (9.88–13.66)^ab^		1.79 (1.97–2.13)^a^		11.83 (10.86–12.79)^ab^	
**Religious faith**										
Muslim	9.81 (9.44–10.19)^a^	0.37	9.68 (9.38–9.98)^a^	0.96	10.41 (10.07–10.75)^a^	9.12^***^	1.95 (1.86–2.03)^a^	7.97^***^	11.52 (11.30–11.75)^a^	2.91
Christian	10.17 (9.26–11.07)^a^		9.99 (9.38–10.61)^a^		11.72 (10.83–12.61)^b^		2.20 (1.98–2.42)^b^		12.07 (11.63–12.51)^a^	
Other (Sikh, Jews, Buddhist, Hindu, other)	10.06 (9.18–10.94)^a^		9.33 (8.63–10.04)^a^		11.95 (11.11–12.78)^b^		2.35 (2.12–2.57)^b^		11.98 (11.50–12.45)^a^	
**Employment**										
Paid employment	10.27 (9.85–10.69)^a^	4.48^*^	9.73 (9.59–10.24)^a^	4.01^*^	11.24 (10.87–11.62)^a^	13.07^***^	2.12 (2.02–2.22)^a^	4.05^*^	11.79 (11.55–12.03)^a^	3.96^*^
Other	9.54 (9.03–10.06)^b^		8.31 (8.99–9.79)^b^		10.10 (9.60–10.60)^b^		1.96 (1.84–2.08)^b^		11.39 (11.08–11.70)^b^	
**Change in Employment**										
No change	10.41 (9.74–11.08)^a^	0.66	9.41 (9.32–10.34)^a^	0.01	11.17 (10.88–12.18)^a^	0.82	2.35 (2.17–2.52)^a^	13.93^***^	11.92 (11.17–12.03)^a^	1.62
Any change	10.05 (9.53–10.57)^a^		10.04 (9.39–10.19)^a^		11.53 (10.72–11.62)^a^		1.97 (1.85–2.08)^b^		11.60 (11.64–12.19)^a^	
**Living status**										
Living alone	11.55 (10.35–12.76)^a^	4.79^**^	9.55 (8.71–10.39)^a^	5.07^**^	9.92 (8.82–11.03)^a^	2.05	2.13 (1.86–2.39)^a^	0.61	11.80 (11.16–12.44)^a^	0.92
Living with immediate family	9.78 (9.42–10.13)^b^		9.62 (9.33–9.89)^a^		10.96 (10.63–11.30)^a^		2.05 (1.96–2.13)^a^		11.67 (11.47–11.88)^a^	
Living with extended family	10.07 (9.00–11.14)^ab^		10.93 (10.13–11.74)^b^		10.57 (9.75–11.39)^a^		2.17 (1.96–2.34)^a^		11.28 (10.63–11.92)^a^	
**Chronic health conditions**	
Yes	9.04 (8.15–9.93)^a^	4.81^*^	9.79 (9.10–10.48)^a^	0.08	11.77 (10.96–12.58)^a^	6.21^**^	2.17 (1.96–2.38)^a^	1.71	11.41 (10.88–11.95)^a^	1.11
No	10.07 (9.72–10.41)^b^		9.67 (9.42–9.95)^a^		10.66 (10.34–10.98)^b^		2.02 (1.94–2.10)^a^		11.70 (11.50–11.90)^a^	
**Live with someone with chronic health condition**										
Yes	8.90 (8.32–9.47)^a^	9.88^***^	9.52 (8.98–10.06)^a^	0.65	10.45 (9.89–11.01)^a^	3.06	2.08 (1.94–2.22)^a^	0.57	11.56 (11.15–11.96)^a^	0.08
No	10.07 (9.67–10.48)^b^		9.76 (9.46–10.07)^a^		11.08 (10.69–11.46)^a^		2.01 (1.91–2.11)^a^		11.63 (11.40–11.86)^a^	

Means sharing the same superscript letter are not significantly different from each other (p < 0.05), CI, confidence interval; ^*^p < 0.05, ^**^p < 0.01, ^***^p < 0.001.

Use of mainstream sources were found to vary by participants age [*F*_(4, 843)_ = 8.26, *p* < 0.001], ethnicity [*F*_(4, 890)_ = 4.43, *p* = 0.002], religion [*F*_(2, 844)_ = 9.12, *p* < 0.001] and employment status [*F*_(1, 847)_ = 13.07, *p* < 0.001]. Information accessed through this source was significantly higher among older (51 years+) participants, those in paid employment, and among those who do not self-identify as Muslim. In addition, participants who were Indian alongside those with a chronic health condition [*F*_(1, 849)_ = 6.21 *p* = 0.013] were found to be significantly more likely to access health information through mainstream sources.

Participants' access to health information through healthcare professionals varied depending on religion [F(2,872) = 7.98, *p* < 0.001], education level [F(3,827) = 4.65, *p* = 0.003], current employment status [F(1,875) = 4.05, *p* = 0.044] and change in employment status [F(1,572) = 13.93, *p* < 0.001]. The findings confirmed that those who ascribed as non-Muslim, have a higher education level (university education or above) alongside those who are either in paid employment and/or have not had a change to their employment status since the pandemic started were all significantly more likely to access their information through healthcare professionals.

Use of social networks as sources of information about COIVID-19 varied by participants' employment [*F*_(1, 857)_ = 33.30, *p* < 0.001] and living status [*F*_(2, 853)_ = 4.77, *p* = 0.009]. The findings confirmed that participants who live with their extended family alongside those not in paid employment were significantly more likely to access COVID-19 related information through their social networks.

In summary, the findings highlight that level of education and employment status greatly influenced the participants usage of sources to access health information, with those in paid employment and University level of education or above more likely to seek information through social media, mainstream, and healthcare professionals. Older participants whilst less likely to seek health information through social media platforms were revealed to be more likely to use mainstream sources. Those who live with extended family and/or not in paid employment were most likely to use their existing social networks to source information related to COVID-19.

Participants were also asked to rate to what extent they trusted the sources to provide accurate information about COVID-19. Interestingly, whilst doctors and/or healthcare providers appeared to be one of the least accessed, these sources were cited as the most trusted for gaining information closely followed by health apps (Table 5).

**Table 5:** Trustworthiness of sources to provide accurate information about COVID-19.

**Items**	**Not at all**	**Somewhat**	**Mostly**	**Completely**
	***N*** **(%)**	***N*** **(%)**	***N*** **(%)**	***N*** **(%)**
Twitter	453 (51.2)	322 (36.4)	90 (10.2)	19 (2.1)
Facebook	450 (49.8)	355 (39.3)	84 (9.3)	1 (1.6)
Instagram	436 (48.6)	363 (40.5)	83 (9.3)	15 (1.7)
WhatsApp	356 (39.3)	390 (43.0)	135 (14.9)	25 (2.8)
Newspaper (National)	174 (19.3)	358 (39.7)	307 (34.0)	63 (7.0)
Newspaper (Local)	186 (20.6)	358 (39.7)	292 (32.3)	65 (7.2)
Family members	132 (14.5)	451 (49.5)	235 (25.8)	93 (10.2)
Friends	132 (14.6)	476 (52.7)	216 (23.9)	80 (8.8)
Co-workers	162 (18.1)	450 (50.2)	211 (23.5)	73 (8.1)
Doctors/ healthcare professionals	73 (8.1)	170 (18.8)	357 (39.6)	302 (33.5)
National radio	174 (19.6)	361 (40.7)	263 (29.7)	88 (9.9)
Local community radio	181 (20.5)	346 (39.1)	267 (30.2)	90 (10.2)
National TV	115 (12.6)	312 (34.2)	340 (37.2)	146 (16.0)
Local TV	133 (14.9)	340 (38.0)	299 (33.4)	123 (13.7)
Health Apps (NHS, other)	104 (11.5)	208 (22.9)	315 (34.7)	281 (30.9)

We also asked participants to comment on the extent to which they determine the accuracy of the sources that they access information relating to COVID-19 (Supplementary Table 5). The extent to which participants validated the sources they used to access information were shown to vary by education [*F*_(3, 853)_ = 7.91 *p* < 0.001] and employment status [*F*_(1, 904)_ = 3.96 *p* = 0.047] (Table 4). The findings confirmed that participants with no formal qualifications and those not in paid employment were significantly less likely to validate the sources that they access for COVID-19 related information.

### 3.4. Perceptions of risk factors for COVID-19

Participants rated the extent to which they agreed that provided risk factors contributed to placing ethnically diverse populations at increased risk of contracting COVID-19. The highest scored risk factors included types of employment (e.g., working in front-line health and social care roles, working in public-facing services) (*M* = 4.30; SD = 1.05), living in overcrowded accommodation (*M* = 4.21; SD = 1.02) and living in densely populated areas (*M* = 4.15; SD = 1.05). The least cited reason was identified as having low levels of English literacy and proficiency (*M* = 3.18; SD = 1.36) (Supplementary Table 6).

Participants were also asked to rate the extent to which they agreed that several provided risk factors were contributory factors in putting ethnically diverse populations at risk of poorer health outcomes as a consequence of being diagnosed with COVID-19. The highest-rated risk factor was revealed to be having a pre-existing health condition (such as diabetes, hypertension, cardiovascular disease, and obesity) (*M* = 4.36; SD = 0.99), with the lowest common reasons including; low levels of English literacy and proficiency (*M* = 3.20; SD = 1.30) and low income or financial insecurity (*M* = 3.41; SD = 1.24) (Supplementary Table 7).

### 3.5. Perceived likelihood of being negatively affected by COVID-19

The total score of the perceived risk related to COVID-19 events among the participants ranged from 0 to 48 with a mean of 28.92 (SD = 7.23) (Table 6).

**Table 6:** Perceived risk of event likelihood related to COVID-19.

**Items**	**Extremely unlikely**	**Moderately unlikely**	**Neither likely nor unlikely**	**Moderately likely**	**Extremely likely**	**Already happened**
	***N* (%)**	***N* (%)**	***N* (%)**	***N* (%)**	***N* (%)**	***N* (%)**
You will be infected	56 (5.9)	98 (10.4)	165 (17.4)	435 (46.0)	160 (16.9)	32 (3.4)
Someone in your family will be infected	49 (5.1)	89 (9.3)	133 (13.9)	436 (45.6)	205 (21.2)	53 (5.5)
One of your friends will be infected	36 (3.7)	63 (6.6)	128 (13.5)	453 (47.8)	196 (20.7)	72 (7.6)
One of your colleagues will be infected	61 (6.4)	57 (6.0)	148 (15.7)	416 (44.1)	202 (21.4)	60 (6.4)
You will have to go to the hospital if you get infected	93 (9.1)	178 (18.8)	198 (20.9)	343 (36.2)	129 (13.6)	6 (0.1)
You will have to go into quarantine independent of being infected or not	70 (7.4)	88 (9.3)	133 (14.0)	353 (37.3)	269 (28.4)	34 (3.6)
You will get infected, and you will infect someone else	62 (6.5)	111 (11.8)	157 (16.6)	399 (42.3)	191 (20.3)	23 (2.4)
Someone in your direct environment (family, friends, colleagues) will die	104 (11.7)	133 (14.2)	233 (24.9)	306 (32.7)	115 (12.3)	40 (4.3)

Participants perceived susceptibility varied by age [*F*_(4, 936)_ = 6.16, *p* < 0.001], ethnicity [*F*_(4, 926)_ = 3.52, *p* = 0.007], religion [*F*_(2, 938)_ = 9.02, *p* < 0.001] and employment status [*F*_(1, 938)_ = 8.28, *p* = 0.004]. Differences were also found for participants' living status [*F*_(2, 939)_ = 8.29, *p* < 0.001] and among those who have [*F*_(1, 947)_ = 7.87, *p* = 0.005] or live with someone who has a chronic health condition [*F*_(1, 947)_ = 7.87, *p* = 0.005] (Table 2). The findings confirmed that participants who were older (41 years and older), in paid employment, Muslim and/or have or live with someone with a chronic health condition had significantly increased levels of perceived risk. However, in contrast participants who identified as black African/Caribbean alongside those who live alone were found to have significantly lower levels of perceived risk.

### 3.6. Attitudes toward the effectiveness of health-protective actions

We assessed participants' attitudes toward the effectiveness of health-protective actions with the items presented. This says ‘Participants' scores ranged from 13 to 45 with a mean of 36.15 (SD = 6.67) (Table 7). The correlations are shown in Table 8 clearly indicate strong relationships between all of the predictor factors (knowledge, perceived risk) and attitudes toward the effectiveness of health-protective actions.

**Table 7:** Attitudes toward the effectiveness of protective measures to keeping you safe from COVID-19.

**Items**	**Extremely effective**	**Very effective**	**Moderately effective**	**Slightly effective**	**Not effective at all**
	***N*** **(%)**	***N*** **(%)**	***N*** **(%)**	***N*** **(%)**	***N*** **(%)**
Wearing a facemask	433 (45.3)	268 (28.0)	146 (15.3)	77 (8.1)	32 (3.3)
Washing your hands frequently	587 (61.5)	232 (24.3)	88 (9.2)	41 (4.3)	7 (0.7)
Seeing or speaking to a health care professional if you feel sick	265 (28.2)	238 (25.3)	277 (29.4)	99 (10.5)	62 (6.6)
Seeing or speaking to a healthcare professional if you feel healthy although concerned you were exposed	233 (24.7)	208 (22.0)	265 (28.1)	143 (15.1)	95 (10.1)
Avoiding public spaces, gatherings, and crowds	541 (56.8)	244 (25.6)	100 (10.5)	49 (5.1)	19 (2.0)
Avoiding contact with those who could be at high risk	588 (61.9)	225 (23.7)	80 (8.4)	38 (4.0)	19 (2.0)
Avoiding hospitals and clinics	387 (40.9)	242 (25.6)	194 (20.5)	76 (8.0)	48 (5.1)
Avoiding restaurants	442 (46.7)	235 (24.8)	162 (17.1)	77 (8.1)	31 (3.3)
Avoiding public transport	452 (47.7)	235 (24.8)	166 (17.5)	70 (7.4)	25 (2.6)

**Table 8:** Correlations and descriptive statistics for attitudes toward effectiveness of protective measures and predictor variables.

**Variable**	**1**	**2**	**3**	**Mean**	**SD**
1. Knowledge (Symptoms)	–			9.45	5.08
2. Knowledge (Transmission routes)	0.38^***^	–		2.48	0.93
3. Perceived risk	0.14^***^	0.10^**^	–	28.92	7.23
4. Attitudes toward effectiveness of protective measures	0.07^*^	0.12^***^	0.23^***^	36.15	6.67

^*^p < 0.05; ^**^p < 0.01; ^***^p < 0.001.

The results confirmed that for attitudes toward COVID-19 health-protective significant differences were found for age [*F*_(4, 894)_ = 3.08, *p* = 0.016], ethnicity [*F*_(1, 4)_ = 3.04, *p* = 0.017], living status [*F*_(2, 896)_ = 3.51, *p* = 0.030], and among those who disclosed that they had a chronic health condition [*F*_(1, 798)_ = 4.27, *p* = 0.039] (Table 2). Participants who live either alone or with someone with an existing health condition alongside those who self-identified as black African/Caribbean were significantly less likely to have positive attitudes toward health-protective actions.

A hierarchical multiple regression analysis was run to determine whether participant's socio-demographic factors (age, ethnicity, living status), living with someone with a chronic disease, knowledge of COVID-19 symptoms and transmission routes and perceived levels of susceptibility toward COVID-19 could predict their attitudes toward COVID-19 protective measures.

The results as shown in Table 9 revealed that the background variables (age, ethnicity, living status, living with someone with chronic disease) explained 4% of the variance of attitudes toward health-protective actions which was significant *R*^2^ = 0.04, *F*_(8, 741)_ = 3.54, *p* < 0.001 (model 1). Knowledge (symptoms and transmission routes) added a significant increase of 1% of additional variance *F*_(2, 739)_ = 5.89, *R*^2^ change =0.02, *p* = 0.003 (model 2). Perceived susceptibility also added a significant increase of 4% of the variance *F*_(1, 738)_ = 29.42, *R*^2^ change = 0.04, *p* < 0.001 (model 3).

**Table 9:** Hierarchical regression of variables predicting attitudes toward COVID-19 protective measures.

**Variable**	**Model 1**			**Model 2**			**Model 3**		
	***B***	***SE B***	β	***B***	***SE B***	β	***B***	***SE B***	β
Age	0.05	0.02	0.12^**^	0.05	0.02	0.10^**^	0.037	0.02	0.08^*^
Indian	3.02	1.55	0.16^*^	3.25	1.54	0.17^*^	3.64	1.52	0.19^*^
Pakistani	2.82	1.50	0.17	1.92	1.43	0.14	2.10	1.41	0.16
Bangladeshi	1.67	1.44	0.13	3.00	1.49	0.18^*^	3.41	1.47	0.20^*^
Black African/Caribbean	0.56	1.57	0.03	0.83	1.57	0.04	1.46	1.54	0.07
Live alone	1.43	6.57	0.01	1.22	6.53	0.01	2.48	6.41	0.01
Live with extended family	1.41	0.76	0.07	1.55	0.75	0.08^*^	1.60	0.74	0.08^*^
Live with someone with chronic disease	0.67	0.55	0.05	0.64	0.54	0.04	0.50	0.53	0.03
Knowledge (symptoms)				0.08	0.05	0.05	0.05	0.05	0.03
Knowledge (transmission route)				0.99	0.34	0.11^**^	0.89	0.33	0.10^**^
Susceptibility							0.19	0.04	0.20^***^
*R* ^2^	0.04^***^			0.05^***^			0.09^***^		
Δ*R*^2^**=**	–			0.01^**^			0.04^***^		

^*^p < 0.05 ^**^p < 0.01 ^***^p < 0.001.

The combination of all the predictors considered accounted for 9% of the variance, *R*^2^ = 0.09, F(11,738) = 6.49, *p* < 0.001. Of all the factors considered, age (β = 0.08, *p* = 0.025), being Indian (β = 0.19, *p* = 0.017), Bangladeshi (β = 0.20, *p* = 0.020), living with extended family (β = 0.08, *p* = 0.030), knowledge of transmission routes (β = 0.10, *p* = 0.006), and susceptibility (β = 0.20, *p* < 0.001) were significant predictors of attitudes toward COVID-19 health-protective actions (Table 9).

## 4. Discussion

The results provide an increased understanding of the knowledge, perceived risk, and attitudes towards COVID-19 protective measures among an ethnically diverse community located in the Southeast of England.

In this study, we examined levels of knowledge regarding symptoms of COVID-19 and routes of transmission. The findings revealed that whilst participants had a good general awareness of the main symptoms of COVID-19 there was notably less awareness of less common and more serious symptoms. Knowledge of the different transmission routes of COVID-19 was shown to vary. Participants who are currently in paid employment, have a higher level of education had significantly increased awareness of the transmission routes. This finding reinforces the importance of increasing health literacy as an intervention to reduce inequalities to ensure that risk communication can reach all population groups irrespective of education level (39).

We examined how information related to the pandemic was accessed. The findings revealed, similar to previously reported studies, that the most used sources to access COVID-19 related information included national TV and social networks (17, 40). In support of previous research (41) social media platforms, particularly Facebook and WhatsApp were also found to be popular sources, particularly among younger populations, those who reside alone alongside those in paid employment and those with higher education status. Mainstream sources such as local and national TV and radio were found to be accessed more frequently by older participants and participants who identify themselves as non-Muslim. Social media can provide a cheap and easily accessible source of health information across populations which and has the potential to provide intelligent real time data to monitor populations about attitudes toward the pandemic (42). However, it is important to acknowledge that relying on these methods could create disproportionate access to health information, particularly among older and less educated sections of the population (43). These findings provides support a useful insight into how to best target health communication campaigns to ensure that they reach all sections of the wider community.

This study also revealed that whilst healthcare professionals were viewed as the most trusted sources for accessing health information about COVID-19; these were identified as the least used. The findings also confirmed that healthcare professionals were more likely to be accessed by those with higher education levels alongside those in paid employment. This could suggest that some sections of the population are either unaware or have limited access to healthcare professionals for obtaining COVID-19 related advice. The trust of information sources particularly in a pandemic has been identified as extremely important, shown to influence how risk communication messages are interpreted and acted on (44, 45). This finding, therefore, reinforces the importance of using “trusted” medical sources such as healthcare professionals and NHS agencies as a useful route to deliver key messages within local communities.

We were also interested in the extent to which participants validate sources that they access information relating to COVID-19. The findings revealed that those with higher education status (i.e., A level equivalent or above) and those in paid employment were significantly more likely to verify sources. These findings are in line with previous research which considers the negative role of socioeconomic disadvantage on accessing information from trusted, validated and accurate sources (41, 46).

This study attempted to uncover the reasons participants believed minority ethnic populations were at an increased risk of contracting COVID-19 and having poorer health outcomes as a consequence of contracting COVID-19. Types of employment (e.g., working in front-line health and social care roles, working in public-facing services), living in overcrowded accommodation, and living in densely populated areas were viewed as the most important factors that explained why ethnically diverse populations are at increased risk of contracting COVID-19. Poorer health outcomes were viewed to be related to having a pre-existing health condition.

Perceived risk related to COVID-19 was shown to be significantly higher in older participants (aged 41–65) those in paid employment alongside those who either have a chronic health condition or live with immediate or extended family. The results also revealed ethnic differences with black Africans shown to hold significantly lower levels of perceived risk compared to all other ethnic groups. This finding supports international evidence which has identified perceived COVID-19 risk to be notably lower among black ethnic communities (47, 48). Perceived risk alongside other factors studied significantly predicted attitudes towards COVID-19 protective measures; however, the amount of variance explained is modest thus more research is needed. In line with previous research perceived susceptibility has been shown to be important predictor in motivating individuals to take health precautions to prevent the spread of disease (49). This, therefore, highlights the importance of targeted health communication strategies across high-risk populations groups which outline the representative burden of health outcomes in relation to COVID-19 (47, 50, 51).

### 4.1. Strengths and limitations

There is a growing body of international literature which has examined knowledge, attitudes, and perceptions toward the COVID-19 pandemic. However, whilst this provides useful evidence most of these studies have recruited participants through online platforms (27, 52, 53). This can limit the engagement of underserved communities who are unable to read English and/or do not have internet access to participate. This paper represents one of the largest cross-sectional studies to date which has explored knowledge, perceived risk, and attitudes toward COVID-19 among an ethnically diverse population in the UK. The recruitment strategy particularly through the use of community researchers enabled us to achieve a large number of responses (*n* = 1,058) and engage with the less visible sections of the population. Nonetheless, it is important to note that the sample may not be nationally representative of the wider UK population. Data was also not reported on health practises and therefore it was not possible to conclude if participants' attitudes and beliefs explored were predictive of actual behaviour.

### 4.2. Conclusions

In conclusion, this study has contributed to current evidence through understanding the influential factors that could facilitate understanding of risk and promote adherence to health-protective actions among ethnically diverse communities. The findings revealed that there is a wide range of socio-demographic factors that influence the sources that people use to access information about the current pandemic. Those with higher education status and those in paid employment were more likely to authenticate these information sources, which adds to the existing debate of how socioeconomic disadvantage can impact the access to trusted, validated and accurate sources (41, 46). Therefore, improving the local information provision including using tailored communication strategies that draw on trusted sources of the communities including healthcare professionals could facilitate understanding of risk and promote adherence to health-protective actions. The present study also revealed the important influence of socio-demographic factors, health status and perceived risk on attitudes toward protective actions. This is an important finding which signals the need through targeted communication strategies to raise levels of perceived threat among specific sections of the population.

## Data availability statement

The original contributions presented in the study are included in the article/Supplementary material, further inquiries can be directed to the corresponding author.

## Ethics statement

The studies involving human participants were reviewed and approved by University of Bedfordshire. Written informed consent from the participants' legal guardian/next of kin was not required to participate in this study in accordance with the national legislation and the institutional requirements.

## Author contributions

EC, NA, MW, and GR conceived and designed the study. IN, MW, NA, and EC collected the data with support of EE, SC, and CE. EC, LD, AGu, and AGa analysed the data. EC wrote the first draught of the manuscript. EC, NA, GR, LD, AGu, and AGa contributed to revisions of the manuscript. All authors approved the final manuscript.
