# Supplementary material for: Knowledge, perceived risk, and attitudes towards COVID-19 protective measures amongst ethnic minorities in the UK: A cross-sectional study
Source: Front Public Health. 2023 Jan 13;10:1060694. doi: 10.3389/fpubh.2022.1060694 (PMC9880421; doi:10.3389/fpubh.2022.1060694)
Supplement: Supplementary file 1 [file Data_Sheet_1.PDF]

Supplementary Material

Section 1: About you

We would like to start by asking some questions about you and your background.

Q1: What is your age? ______________________

Q2: What is your gender? *Please select one option only*

☐Male
☐Female
☐Transgender
☐Non-binary
☐Any other gender identity (*please specify)* ______________________

Q3: What is your ethnic group? *Please select one option only/ethnic group that you self-identify with.*

Asian/Asian British:
☐Indian
☐Pakistani
☐Bangladeshi

Black/African/Caribbean/black British:
☐African
☐Caribbean

Mixed ethnic background:
☐White and black Caribbean
☐White and black African
☐White and Asian

☐Any other ethnic background *(please specify)* ______________________

Q4: What is your religion? *Please select one option only*

☐No religion
☐Christian (inc. CofE, Catholic, Protestant and other Christian denominations)
☐Buddhist
☐Hindu
☐Jewish
☐Muslim
☐Sikh
☐Any other religion (*please specify)* ______________________

Q5: Who in your family was born in the UK?

You	Yes ☐	No ☐
Your parents	Yes ☐	No ☐
Your grandparents	Yes ☐	No ☐

Q6: What is the highest level of education you have completed? *Please select one option only*

☐No formal qualifications
☐GCSE or equivalent
☐A-Level or equivalent
☐First Degree (e.g., BSc, BA)
☐Higher degree (e.g., MSc, MA)
☐Other (*please specify*) ______

Q7: Which of the following best describes your employment <u>before the COVID-19 pandemic (before March 1st, 2020)?</u> *Please select only one option*

☐Employed full time (35+ hours per week)
☐Employed part-time (less than 35 hours per week
☐Self-employed
☐Full time student
☐Part time student
☐Unemployed
☐Unable to work for health reasons
☐Stay at home parent/carer
☐Unemployed
☐Retired
☐Other (*please specify*) ______

Q8: How has your employment status changed <u>since the COVID-19 pandemic (after March 1st, 2020)?</u> *Please select only one option*

☐I am still going to my workplace for the same number of hours as before the COVID-19 pandemic
☐I am still going to my workplace but am working reduced hours
☐I am working from home
☐I have been furloughed
☐I have lost my job
☐I had to quit my job because of caring responsibilities (children, parents)
☐Other (*please specify*) ______

Q9: What best describes your living arrangements? *Please select only one option*

☐Living alone
☐Living with immediate family
☐Living with extended family
☐Other (*please specify*) ______________________

Q10: Do you currently live with a chronic disease (including chronic lung disease, diabetes, cardiovascular disease, chronic renal or liver disease)?

☐Yes
☐No

Q11: Does anyone in your household, other than you, live with a chronic disease (including chronic lung disease, diabetes, cardiovascular disease, chronic renal or liver disease)?

☐Yes
☐No

Q12: Please provide your postcode ______________________

Section 2: Views about COVID-19

We want to ask you some questions about your awareness and attitudes towards COVID-19.

Q13: From the list below, select those which you think are symptoms of COVID-19? Please tick whichever is appropriate. You can select more than one option.

Sore throat	
Fever	
Cough	
Runny nose	
Shortness of breath at rest	
Shortness of breath when moving (like walking upstairs)	
Chills	
General lack of energy or fatigue	
Loss of appetite	
Discomfort, tightness, or pressure in chest	
Vomiting	
Nausea	
Muscle aches	
Joint aches	
Headache/s	
Seizure/s	
Dizziness	
Altered consciousness or feeling like it is difficult to stay awake	
Loss of ability to smell	
Loss of ability to taste	

Q14: From the list below, select those where you think COVID-19 can be transmitted? *Please tick whichever is appropriate. You can select more than one option.*

Through close contact with an infected person who has symptoms	
Through close contact with an infected person even if they are not showing symptoms of infection	
Through contact with surfaces an infected person has touched	

Q15: To what extent do you agree that the reasons below contribute to increasing the risk of contracting COVID-19 among people from Pakistani, Bangladeshi, Indian, black Caribbean, black African or mixed heritage ethnic background.

	Strongly disagree	Somewhat disagree	Neither agree nor disagree	Somewhat agree	Strongly agree
Types of employment					
Lack of confidence to raise concerns about safety in the workplace					
Use of public transport to get to work/other					
Living in more densely populated areas					
Low income or financial insecurity					
Living in overcrowded accommodation					
Living in multi-generational housing					
Individual behaviours e.g., social distancing, wearing a facemask					
Lack of education on reducing personal risk					
Low levels of English literacy and proficiency					

Q16: To what extent do you agree that the reasons below reasons contribute to putting people from Pakistani, Bangladeshi, Indian, black Caribbean, black African or mixed heritage ethnic backgrounds at an increased risk of poorer health outcomes including being admitted to hospital, being put on a ventilator to support breathing or dying of COVID-19

	Strongly disagree	Somewhat disagree	Neither agree nor disagree	Somewhat agree	Strongly agree
Having a pre-existing health condition					
Mental health issues/illness					
Poor access to healthcare services					
Lack of trust of NHS services and health care treatment					
Stigma relating to being tested and receiving treatment					
Low income or financial insecurity					
Living in areas with high deprivation					
Vitamin D deficiency					
Lack of education and understanding about symptom recognition and when to access health services for COVID 19					
Low levels of English literacy and proficiency					

Q17: How likely do you think the following events will happen in light of the current COVID-19 pandemic?

	Not relevant	Extremely unlikely	Moderately unlikely	Neither likely nor unlikely	Moderately likely	Extremely likely	Already happened
You will be infected							
Someone in your family will be infected							
One of your friends will be infected							
One of your colleagues will be infected							
You will have to go to the hospital if you get infected							
You will have to go into quarantine independent of you being infected or not							
You will get infected and you will infect someone else							
Someone in your direct environment (family, friends, colleagues) will die							

Q18: In your opinion, how effective are the following actions for keeping you safe from COVID-19?

	Extremely effective	Very effective	Moderately effective	Slightly effective	Not effective at all
Wearing a face mask					
Washing your hands with soap or using hand sanitiser frequently					
Seeing or speaking to a healthcare provider if you feel sick					
Seeing or speaking to a healthcare professional if you feel healthy but worry that you were exposed					
Avoiding public spaces, gatherings, and crowds					
Avoiding contact with people who could be at high risk					
Avoiding hospitals and clinics					
Avoiding restaurants					
Avoiding public transport					

Section 3: Getting information about COVID-19

These questions ask you about the sources where you receive information about COVID-19 and whether you think these sources are trustworthy.

Q19: Do you have a computer at the place you live that you can use?

☐Yes
☐No

Q20: Do you currently own a smartphone (a phone that also has computer capabilities and access to the internet)?

☐Yes
☐No

Q21: How often do you access the following sources to get information about COVID-19?

	Multiple times per day	Daily	Weekly	Monthly	Never
Twitter					
Facebook					
Instagram					
WhatsApp					
National Newspaper					
Local Newspaper					
Family members					
Friends					
Co-workers					
Doctors/healthcare providers					
National radio					
Local community radio					
Local TV					
National TV					
Health apps (NHS, other)					

Q22: How much do you trust the following sources to provide accurate COVID-19 information?

	Not at all	Somewhat	Mostly	Completely
Twitter				
Facebook				
Instagram				
WhatsApp				
National Newspaper				
Local Newspaper				
Family members				
Friends				
Co-workers				
Doctors/healthcare providers				
National radio				
Local community radio				
Local TV				
National TV				
Health apps (NHS, other)				

Q23: How do you ensure that the information you have accessed about COVID-19 is trustworthy?

	Strongly disagree	Disagree	Neither agree or disagree	Agree	Strongly agree
I ensure that information I access is from a trusted reputable well-known source.					
I compare information I access with other reliable sources to ensure it is accurate.					
I access information objectively to determine the information I read is presented in a balanced, reasonable and unbiased manner					

________________________End of Survey__________________________
