# Supplementary material for: Knowledge, perceived risk, and attitudes towards COVID-19 protective measures amongst ethnic minorities in the UK: A cross-sectional study
Source: Front Public Health. 2023 Jan 13;10:1060694. doi: 10.3389/fpubh.2022.1060694 (PMC9880421; doi:10.3389/fpubh.2022.1060694)
Supplement: Supplementary file 2 [file Table_2.DOCX]

Supplementary Material

#### ****Table S2. Participants’ knowledge of symptoms of COVID-19.****

| Items | Yes | No |
| --- | --- | --- |
|  | N (%) | N (%) |
| **Sore throat** | 600 (56.7) | 458 (43.3) |
| **Fever** | 900 (85.1) | 158 (14.9) |
| **Cough** | 909 (85.9) | 149 (14.1) |
| **Runny nose** | 306 (28.9) | 752 (71.1) |
| **Shortness of breath at rest** | 727 (68.7) | 331 (31.3) |
| **Shortness of breath when moving (like walking upstairs)** | 512 (48.4) | 546 (51.6) |
| **Chills** | 379 (35.8) | 679 (64.2) |
| **General lack of energy or fatigue** | 548 (51.8) | 510 (48.2) |
| **Loss of appetite** | 470 (44.4) | 588 (55.6) |
| **Nausea** | 256 (24.2) | 802 (75.8) |
| **Vomiting** | 225 (21.3) | 833 (78.7) |
| **Discomfort, tightness, or pressure in chest** | 457 (43.2) | 601 (56.8) |
| **Muscle aches** | 515 (48.7) | 543 (51.3) |
| **Joint aches** | 407 (38.5) | 651 (61.5) |
| **Headaches** | 482 (45.6) | 576 (54.4) |
| **Seizures** | 94 (8.9) | 964 (91.1) |
| **Dizziness** | 199 (18.8) | 859 (81.2) |
| **Altered consciousness or feeling like it is difficult to stay awake** | 198 (18.7) | 860 (81.3) |
| **Loss of ability to smell** | 890 (84.1) | 168 (15.9) |
| **Loss of ability to taste** | 921 (87.1) | 137 (12.9) |
