# Supplementary material for: Knowledge, perceived risk, and attitudes towards COVID-19 protective measures amongst ethnic minorities in the UK: A cross-sectional study
Source: Front Public Health. 2023 Jan 13;10:1060694. doi: 10.3389/fpubh.2022.1060694 (PMC9880421; doi:10.3389/fpubh.2022.1060694)
Supplement: Supplementary file 3 [file Table_3.DOCX]

Supplementary Material

#### Table S3. Participants’ knowledge of transmission routes of COVID-19.

| Items | Yes | No |
| --- | --- | --- |
|  | N (%) | N (%) |
| Through close contact with an infected person who has symptoms | 918 (86.8) | 140 (13.2) |
| Through close contact with an infected person even if they are not showing symptoms of infection | 886 (83.7) | 172 (16.3) |
| Through contact with surfaces an infected person has touched | 825 (78.0) | 233 (22.0) |
