# Supplementary material for: Knowledge, perceived risk, and attitudes towards COVID-19 protective measures amongst ethnic minorities in the UK: A cross-sectional study
Source: Front Public Health. 2023 Jan 13;10:1060694. doi: 10.3389/fpubh.2022.1060694 (PMC9880421; doi:10.3389/fpubh.2022.1060694)
Supplement: Supplementary file 4 [file Table_4.DOCX]

Supplementary Material

#### Table S4. Frequency of sources used by participants to access information about COVID-19.

| **Items** | **Multiple times per day** | **Daily** | **Weekly** | **Monthly** | **Never** |
| --- | --- | --- | --- | --- | --- |
|  | N (%) | N (%) | N (%) | N (%) | N (%) |
| **Twitter** | 86 (9.7) | 109 (12.3) | 82 (9.2) | 29 (3.2) | 58.1 (65.5) |
| **Facebook** | 120 (13.3) | 214 (23.3) | 57 (6.3) | 45 (5.0) | 465 (51.6) |
| **Instagram** | 123 (13.7) | 202 (22.5) | 68 (7.6) | 38 (4.2) | 467 (52.0) |
| **WhatsApp** | 260 (28.4) | 295 (32.2) | 60 (6.5) | 35 (3.8) | 267 (29.1) |
| **Newspaper (National)** | 45 (5.0) | 176 (19.6) | 116 (12.9) | 61 (6.8) | 499 (55.6) |
| **Newspaper (Local)** | 34 (3.9) | 118 (13.4) | 135 (15.3) | 75 (8.5) | 519 (58.9) |
| **Family members** | 159 (17.5) | 341 (37.4) | 193 (21.2) | 58 (6.4) | 160 (17.6) |
| **Friends** | 152 (16.6) | 306 (33.5) | 206 (22.5) | 88 (9.6) | 162 (17.7) |
| **Co-workers** | 100 (11.3) | 239 (26.9) | 169 (19.1) | 64 (7.2) | 315 (35.5) |
| **Doctors/ healthcare professionals** | 41 (4.6) | 75 (8.4) | 147 (16.5) | 257 (28.8) | 373 (41.8) |
| **National radio** | 60 (6.7) | 183 (20.5) | 128 (14.3) | 71 (8.0) | 451 (50.5) |
| **Local community radio** | 56 (6.3) | 185 (20.8) | 121 (13.6) | 65 (7.3) | 463 (52.0) |
| **National TV** | 176 (19.3) | 377 (41.4) | 145 (15.9) | 56 (6.1) | 157 (17.2) |
| **Local TV** | 137 (15.2) | 325 (36.1) | 145 (16.1) | 49 (5.4) | 245 (27.2) |
| **Health Apps (NHS, other)** | 70 (7.7) | 159 (17.6) | 154 (17.0) | 139 (15.4) | 382 (42.3) |
