# Supplementary material for: Knowledge, perceived risk, and attitudes towards COVID-19 protective measures amongst ethnic minorities in the UK: A cross-sectional study
Source: Front Public Health. 2023 Jan 13;10:1060694. doi: 10.3389/fpubh.2022.1060694 (PMC9880421; doi:10.3389/fpubh.2022.1060694)
Supplement: Supplementary file 5 [file Table_5.DOCX]

Supplementary Material

#### Table S5. Validation of sources used for gaining information about COVID-19.

| Items | Strongly disagree | Disagree | Neither agree nor disagree | Agree | Strongly agree | M (SD) |
| --- | --- | --- | --- | --- | --- | --- |
|  | N (%) | N (%) | N (%) | N (%) | N (%) |  |
| **Information is accessed from a reputable well-known source** | 53 (5.7) | 26 (2.8) | 133 (14.3) | 386 (41.5) | 333 (35.8) | 3.99 (1.06) |
| I compare information I access with other reliable sources to ensure it is accurate. | 58 (6.3) | 43 (4.6) | 196 (21.2) | 355 (38.4) | 273 (29.5) | 3.80 (1.10) |
| I access information objectively to determine the information I read is presented in a balanced, reasonable, and unbiased manner | 51 (5.5) | 29 (3.1) | 200 (21.5) | 382 (41.5) | 268 (28.8) | 3.85 (1.05) |
