# Supplementary material for: Knowledge, perceived risk, and attitudes towards COVID-19 protective measures amongst ethnic minorities in the UK: A cross-sectional study
Source: Front Public Health. 2023 Jan 13;10:1060694. doi: 10.3389/fpubh.2022.1060694 (PMC9880421; doi:10.3389/fpubh.2022.1060694)
Supplement: Supplementary file 6 [file Table_6.DOCX]

Supplementary Material

#### Table S6. Perceived risk factors that contribute to putting ethnically diverse populations at increased risk of contracting COVID-19.

| Items | Strongly disagree | Somewhat disagree | Neither agree nor disagree | Somewhat agree | Strongly agree | M (SD) |
| --- | --- | --- | --- | --- | --- | --- |
|  | N (%) | N (%) | N (%) | N (%) | N (%) |  |
| Types of employment | 48 (5.0) | 19 (2.0) | 83 (8.6) | 263 (27.2) | 554 (57.3) | 4.30 (1.05) |
| Lack of confidence to raise concerns about safety in workplace | 51 (5.3) | 59 (6.2) | 148 (15.4) | 346 (36.1) | 354 (37.0) | 3.93 (1.12) |
| **Use of public transport to get to work/other** | 40 (4.2) | 40 (4.2) | 157 (16.4) | 338 (35.4) | 381 (39.9) | 4.03 (1.05) |
| Living in more densely populated areas | 37 (3.9) | 41 (4.3) | 121 (12.8) | 293 (30.9) | 456 (48.1) | 4.15 (1.05 |
| Low income or financial insecurity | 122 (12.8) | 90 (9.5) | 225 (23.6) | 235 (24.7) | 280 (29.4) | 3.48 (1.34) |
| Living in overcrowded accommodation | 34 (3.6) | 40 (4.2) | 96 (10.0) | 312 (32.6) | 474 (49.6) | 4.21 (1.02) |
| Living in multi-generational housing | 57 (6.0) | 49 (5.2) | 158 (16.6) | 292 (30.7) | 394 (41.5) | 3.97 (1.15) |
| **Individual behaviours e.g., social distancing, wearing facemask** | 94 (9.9) | 61 (6.4) | 129 (13.6) | 237 (25.0) | 428 (45.1) | 3.89 (1.31) |
| Lack of education on reducing personal risk | 73 (7.6) | 60 (6.2) | 161 (16.7) | 319 (33.2) | 349 (36.3) | 3.84 (1.20) |
| Low levels of English literacy and proficiency | 169 (17.9) | 107 (11.4) | 237 (25.2) | 244 (25.9) | 185 (19.6) | 3.18 (1.36) |
