# Supplementary material for: Knowledge, perceived risk, and attitudes towards COVID-19 protective measures amongst ethnic minorities in the UK: A cross-sectional study
Source: Front Public Health. 2023 Jan 13;10:1060694. doi: 10.3389/fpubh.2022.1060694 (PMC9880421; doi:10.3389/fpubh.2022.1060694)
Supplement: Supplementary file 7 [file Table_7.DOCX]

Supplementary Material

#### Table S7. Perceived factors that contribute to putting ethnically diverse populations at increased risk of poorer health outcomes of COVID-19.

| Items | Strongly disagree | Somewhat disagree | Neither agree nor disagree | Somewhat agree | Strongly agree | Mean (SD) |
| --- | --- | --- | --- | --- | --- | --- |
|  | N (%) | N (%) | N (%) | N (%) | N (%) |  |
| **Having a pre-existing health condition** | 40 (4.2) | 20 (2.1) | 60 (6.3) | 266 (27.9) | 567 (59.5) | 4.36 (0.99) |
| **Mental health issues/illness** | 76 (8.1) | 9710.3 | 269 (28.5) | 270 (28.6) | 231 (24.5) | 3.51 (1.20) |
| Poor access to healthcare services | 50 (5.3) | 505.3 | 169 (17.9) | 317 (33.6) | 357 (37.9) | 3.93 (1.12) |
| Lack of trust of NHS services and health care treatment | 61 (6.5) | 63 (6.7) | 213 (22.6) | 295 (31.3) | 312 (33.1) | 3.78 (1.17) |
| Stigma relating to being tested and receiving treatment | 58 (6.2) | 77 (8.2) | 230 (24.6) | 312 (33.4) | 257 (27.5) | 3.68 (1.14) |
| Low income or financial insecurity | 96 (10.2) | 107 (11.4) | 259 (27.6) | 269 (28.6) | 209 (22.2) | 3.41 (1.24) |
| **Living in areas with high deprivation** | 61 (6.5) | 56 (6.0) | 201 (21.5) | 318 (34.0) | 298 (31.9) | 3.79 (1.15) |
| Vitamin D deficiency | 77 (8.2) | 70 (7.5) | 243 (26.0) | 293 (31.3) | 253 (27.0) | 3.61 (1.19) |
| Lack of education and understanding about symptom recognition and when to access health services for COVID 19 | 65 (6.9) | 70 (7.0) | 18319.5 | 323 (34.4) | 298 (31.7) | 3.77 (1.18) |
| Low levels of English literacy and proficiency | 147 (15.9) | 105 (11.3) | 243 (26.2) | 277 (29.9) | 155 (16.7) | 3.20 (1.30) |
